# Attitude, Acceptability and Knowledge of HPV Vaccination among Local University Students in Hong Kong

**DOI:** 10.3390/ijerph13050486

**Published:** 2016-05-11

**Authors:** Vico Chung Lim Chiang, Ho Ting Wong, Pui Chun Au Yeung, Yuk Ki Choi, Michelle Sum Yue Fok, Oi In Mak, Hing Yu Wong, Kim Ho Wong, Shui Yan Wong, Yee Shan Wong, Eugene Ying Yeung Wong

**Affiliations:** School of Nursing, the Hong Kong Polytechnic University, Hong Kong, China; htwong@polyu.edu.hk (H.T.W.); 12067255D@connect.polyu.hk (P.C.A.Y.); 12068436D@connect.polyu.hk (Y.K.C.); 12069419D@connect.polyu.hk (M.S.Y.F.); 12066624D@connect.polyu.hk (O.I.M.); 12068183D@connect.polyu.hk (H.Y.W.); 12070319D@connect.polyu.hk (K.H.W.); 12069426D@connect.polyu.hk (S.Y.W.); 12066815D@connect.polyu.hk (Y.S.W.); 12067453D@connect.polyu.hk (E.Y.Y.W.)

**Keywords:** HPV, papillomavirus, undergraduate students, vaccination

## Abstract

The Human Papillomavirus (HPV) vaccine has the great potential to prevent HPV-related infections for millions of women and men worldwide. However, the success of the vaccine is highly dependent on the vaccination rate. Factors influencing the attitudes of undergraduate students towards HPV vaccination should be studied. This is a cross-sectional survey that was conducted to estimate the HPV vaccination rate among undergraduate students in Hong Kong, and to identify the predictors of their attitude towards HPV vaccination. The results showed that the HPV vaccination rate was 13.3%. Factors related to knowledge of vaccination were the main predictors of the students’ attitude towards vaccination (there were seven predictors, with B = 1.36 to 2.30; *p* < 0.05), followed by gender (B = −1.40; *p* < 0.05), acceptable maximum price (B = 0.35; *p* < 0.05), and willingness to receive the HPV vaccine if it can protect against cervical/anal cancer and genital warts (B = −1.90; *p* < 0.001). The regression model that was developed based on the predictors had a moderate effect size (adj-R^2^ = 0.33). To conclude, the HPV vaccination rate among undergraduate students in Hong Kong was low. They should be provided with more active education and activities to promote HPV vaccination to improve their knowledge on the subject.

## 1. Introduction

The Human Papillomavirus (HPV) is one of the main causes of anogenital warts and cervical cancer [1]. It is the eighth most common form of cancer among Hong Kong women, with a prevalence rate of 11.9 per 100,000 women. It is especially common in women aged between 20 and 44, accounting for 7.9% of all cancers in this demographic [2]. HPV is transmissible through sexual contact, including by having vaginal, anal, or oral sex with an infected person [3]. The Centre for Health Protection reported that the prevalence of HPV in Hong Kong is between 7% and 11% for those attending a cervical screening service [4]. Regular cervical screening and HPV vaccination are the usual means for the early detection or prevention of HPV-related diseases.

It is believed that HPV vaccines can effectively protect the recipient against such HPV-related diseases as cervical cancer, vaginal cancer, vulvar cancer, and genital warts [5]. The HPV vaccination rate of women in the USA and Australia is about 37.6% and 32%, respectively [6], but it is lower in Asian countries. Two types of HPV vaccines have been available in Hong Kong since 2009—the quadrivalent and bivalent HPV vaccines. The Center for Disease Control and Prevention has suggested that people can receive the vaccine starting from the age of nine. Females and males can receive the vaccine until the ages of 26 and 21, respectively [7]. Several programs have been carried out in Hong Kong to promote HPV vaccination, such as the “Youth HPV prevention program” organized by the Society of Physicians of Hong Kong. The aim of this program was to raise awareness of HPV and related diseases. The target groups of this program were full-time primary and secondary school students in Hong Kong, aged nine or above who had never before received the HPV vaccination before. Two or three doses of vaccine will be given depending on the age of the participant. Currently, a “2-in-1 vaccine” (HPV type 16 and 18) and a “4-in-1 vaccine” (HPV type 6, 11, 16, and 18) are provided at a price of HK$700 per dose, excluding the doctor’s and nurse’s free [8]. However, among adolescent girls it is only 7.5% [9], while in a more recent study, the vaccination rate for girls under 18 was reported to be even 2.4% [10]. Although Hong Kong adolescent girls have a generally favorable attitude towards HPV vaccination and HPV programs are promoted widely in Hong Kong through different mass media, the vaccination rate is relatively low compared to that in other developed countries [10,11].

### 1.1. Attitude on Vaccination

There are many studies investigating the perceptions of adolescent girls of the risk of contracting diseases related to HPV, the effectiveness and safety of the HPV vaccine, and their intention to receive the HPV vaccination [12]. According to Kwan *et al.* [11], despite the low HPV vaccination rate in Hong Kong, local women and adolescent girls generally held a favorable attitude towards HPV vaccination. The result of a local study targeting adolescent girls aged between 13 and 21 showed that the majority of the participants thought that cervical cancer was a terrible disease and were willing to take action to prevent its occurrence. Although one-third of the participants wanted to be vaccinated against HPV, nearly 60% of the participants were still in the stage of contemplation, and only about 6.7% of them had actually received the vaccination [13]. This suggests that apart from the perceived risk of contracting cervical cancer, there were other factors affecting the decision of adolescent girls to be vaccinated against HPV that require further exploration.

Regarding studies on male attitudes, these have mostly been conducted in foreign countries. One of the studies conducted by Songthap *et al.* investigated the attitudes of male college students in Thailand towards HPV vaccination [14]. The results showed that about 65% of the participating males agreed that it was important to be vaccinated against HPV before becoming sexually active. Both the male and female participants agreed that cervical cancer could cause death in women, and that they should take the vaccination. However, only 25.3% of them were willing to do so. There is no local study on HPV vaccination that specifically targets adolescent males.

### 1.2. The Acceptability of Vaccination

Acceptance of the HPV vaccine might be influenced by factors affecting the decision-making process that individuals go through when deciding whether or not to be vaccinated. Several local studies have consistently suggested that the cost of the HPV vaccines is a major barrier to their acceptance by mothers and female adolescents [9,10,11,15,16]. The median price that mothers were willing to pay for a full-course of vaccination was HK$1000, substantially lower than the current market price [10]. Apart from the concern over cost, for parents the barriers to vaccination include uncertainty over the side effects of the vaccine such as infertility [17,18,19,20], the duration of the effectiveness of the vaccine, the perception that the risk of becoming infected with HPV is low, the perception that there is no immediate need for vaccination, the anticipation of family disapproval, and the fear of experiencing pain during injections [11]. Other factors included the fear that receiving the vaccination would cause daughters to engage in riskier sexual behavior [21,22,23,24,25], and religious proscriptions against engaging in sexual activities before marriage [26]. By contrast, the factors facilitating acceptance of HPV vaccination include perceived support from family and peers, medical assurances of the safety and efficacy of the vaccine [11], and recommendation of the vaccine by professionals [20].

### 1.3. Knowledge of Vaccination

A local study found that young teenage girls aged 13–20 had a limited knowledge of HPV and its relationship with cervical cancer [11]. For example, they had difficulty in understanding the mechanism linking HPV infection with cervical cancer. Another study in Thailand showed that 73.8% of male students and 69.6% of female students aged 12–15 did not know who should be screened for cervical cancer [14]. Furthermore, the study of Bowyer *et al.* in England indicated that more than 50% of girls aged 15–16 had little knowledge about HPV, the vaccine, and the need for future screening, even if they had been vaccinated [27]. Female undergraduate students in Korea and Nigeria were also found to be insufficiently knowledgeable about HPV and vaccination [28,29]. Reimer *et al.* also noted that among whites and Hispanics, males were less aware and knowledgeable about HPV and the HPV vaccine than females [30]. There have been a number of studies focusing on female knowledge of HPV and the vaccine, but a paucity of studies on the knowledge of males.

### 1.4. Research Gaps and Aim/Purpose of the Study

There have been many studies investigating the attitudes of school students and their parents toward HPV vaccination, but research focusing on young adults is limited [11,13,14]. According to a study on youth sexuality conducted in 2011 by The Family Planning Association of Hong Kong, around half of young adults aged between 18 and 27 had sexual experience. Nearly 3% of them had their first experience before the age of 16 [31]. The aim of this study was to explore the perceptions of university students from Hong Kong towards HPV and HPV vaccination, in terms of their attitudes, acceptance, and knowledge. The objectives were to identify the underlying factors that might affect the attitudes of the university students, and to compare the attitudes and knowledge of male and female students on HPV vaccination. The results of the study provided findings and insights that will allow researchers and public health professionals to better promote and continue to conduct research on HPV vaccination for young adults, particularly males; and over a longer term to achieve better control of the diseases related to HPV.

## 2. Materials and Methods

### 2.1. Study Design

This is a cross-sectional survey that was conducted in a major university in Hong Kong from April to September 2015.

### 2.2. Sampling Method

Convenience sampling was conducted to recruit subjects for this study. There was no special event or news related to HPV according to the press releases issued in the study period by the Centre of Health Protection and the Department of Health. The selected university has 18,777 undergraduate students, 13,954 of whom were enrolled in publicly funded programs. These students constitute 17.25% of the total number of publicly funded undergraduate students in Hong Kong. The result of the sample size calculation showed that at least 377 students are required to keep the margin of error within 5% and the confidence level at 95% [32]. A set of questionnaires in paper format was distributed to students and collected during their lectures, while some questionnaires were completed by students randomly selected on campus during school days. The students were given no incentives to participate in the survey.

### 2.3. Inclusion and Exclusion Criteria

The criteria for inclusion were: (1) undergraduate students studying in the selected university; and (2) capable of communicating in English. The criteria for exclusion were: (1) undergraduate students under the age of 18; (2) students from Mainland China and overseas; and (3) undergraduate students 25 years of age or older. Students from Mainland China were excluded because they only comprised around 8% of the undergraduate students in the university [33]. Moreover, from the view of health policy, the priority should be on local students because they are expected to stay and contribute to Hong Kong in the long run.

### 2.4. Instrument

The set of questionnaires used in this study was modified from a study conducted by a Thai research team that was also investigating similar issues among Thai students [14]. The eight-page questionnaire is designed to collect data on the university students in four areas: (1) Basic information on their socio-demographic and medical backgrounds (17 items); (2) Their knowledge of HPV, cervical cancer, and the HPV vaccine (22 items). Each question has three possible options (Yes/No/Don’t know); the option of “don’t know” is considered an incorrect answer; (3) Attitude towards HPV, cervical cancer, and the HPV vaccine (21 items). The items are in a 5-point Likert scale from 1 “strongly disagree” to 5 “strongly agree”; (4) Acceptability of the HPV vaccine (6 items). The questions relate to the students’ preferred place and price for receiving the HPV vaccination, and to their belief of which group of people should be vaccinated against HPV. Overall, the scale-level content validity index of the questionnaire was found to be 0.92, which is an acceptable level [34].

### 2.5. Data Analysis

Descriptive statistics were used to identify a general picture of the demographics and medical backgrounds of the students, and their attitudes, knowledge, and acceptance of HPV vaccination. To compare gender differences in the students’ attitude and knowledge of HPV vaccination, an independent sample *t*-test was adopted because only two groups of students were involved in this cross-sectional study. Finally, through a bivariate analysis, the students’ socio-demographic and medical backgrounds, and their acceptance and knowledge of HPV vaccination were correlated with their total score on their attitude towards HPV vaccination. Potential predictors with a *p*-value of smaller than 0.1 found in the bivariate analysis were further analyzed using multivariate regression analysis, in order to further confirm the significant predictors of the students’ attitude towards HPV vaccination. All of the statistical analyses were conducted using SPSS version 23 (IBM, Armonk, NY, USA). The alpha level was set at 0.05.

### 2.6. Ethical Considerations

Before completing the questionnaires, the subjects were well informed about the purpose and contents of the study. They were given an information sheet and asked to sign a consent form to document their voluntary participation. They were also informed that their participation was totally voluntary and that they had the right and freedom to withdraw from the study at any time without providing a reason. This study was approved by the Human Subjects Ethics Committee of the related university (HSEARS20150326003).

## 3. Results

### 3.1. Response Rate

A total of 520 sets of questionnaires were distributed; 437 sets of completed questionnaires were collected, for a response rate of 84%.

### 3.2. Socio-Demographic Characteristics

Table 1 shows that around half of the subjects were male (46.5%) and enrolled in a healthcare-related program (53.1%). Two-thirds of them were aged 18–21 (66.6%). The majority of the subjects had a monthly income of less than HK$3000 (67.3%). Most of them were living with family (90.4%) and were without any religious beliefs (72.4%). Around two-thirds of them were single (63.2%), while the remaining one-third were in a relationship with one person (36.4%), with the exception of two subjects (0.5%), who were in relationships with more than one person. Only one-fifth of them had sexual experience (17%).

### 3.3. Health-Related Characteristics

The estimated vaccination rate was 13.3%, with the female vaccination rate (24.4%) being significantly higher than that of the male (0.5%). Virtually all of the participants had no history of cervical cancer (100%), genital warts (99.5%), or anal cancer (99.8%). Around 90% of them had heard about cervical cancer (87.9%) and the HPV vaccine (92.2%), with television being the main source of this information (around 75%), followed by the Internet, newspapers/magazines, and their healthcare provider (all slightly more than 40%). More than two-thirds of them stated that they would like to be vaccinated if the HPV vaccine can protect them against cervical/anal cancer and genital warts (69.6%), while one-fifth of them were unwilling to be vaccinated (19.5%) (Table 2).

### 3.4. Acceptability of HPV Vaccination

Near half of the subjects preferred to be vaccinated at the health clinic of a university (43.2%) followed by a public hospital (34.1%). Of the participants, 77.6% believed that both males and females should get the vaccination equally if the HPV vaccine can protect against both cervical cancer and genital warts, while only 22% of them believed that “only females” or “females should get the vaccine rather than males”. If the HPV vaccine is beneficial for adolescents who are not sexually active, most of the respondents believed the ideal age for vaccination to be between the ages of 11 and 18, with 15–16 years old being the age range chosen by the largest percentage of respondents (26.8%) (Table 3).

**Figure 1:**
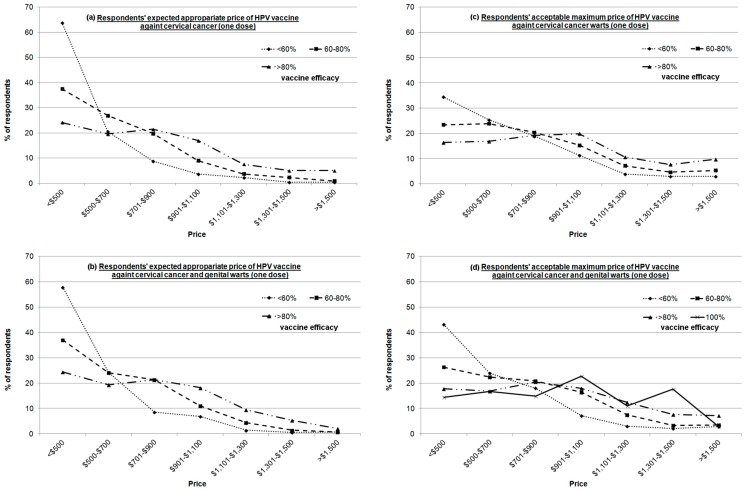
Respondents’ expectations of the appropriate and maximum prices for HPV vaccine. (**a**) Respondents’ expectations of the appropriate price for the HPV vaccine against cervical cancer; (**b**) Respondents’ expectations of the appropriate price for the HPV vaccine against cervical cancer and genital warts. (**c**) Respondents’ acceptable maximum price for the HPV vaccine against cervical cancer; (**d**) Respondents’ acceptable maximum price for the HPV vaccine against cervical cancer and genital warts.

Regarding expectations of the appropriate price for HPV vaccination, most of the respondents chose the lowest price option (<$500). Regardless of the efficacy of the vaccine, the proportion of respondents who indicated that the price was acceptable decreased as the price increased (Figure 1a,b). The pattern was slightly different for the maximum acceptable price. When the efficacy of the vaccine was sufficiently high, the respondents were willing to pay a higher price (Figure 1c,d). For instance, when the efficacy of the vaccine in preventing both cervical cancer and genital warts was 100%, most of the respondents replied that the maximum affordable price was $901–$1100. The proportion of respondents who chose $1301–$1500 was even larger than the group that chose <$500 (Figure 1d).

### 3.5. Attitude towards HPV Vaccination

Regarding the respondents’ attitude towards HPV vaccination, most of the statements received a rating higher than the middle score (*i.e.*, 3), with the exception of the following four statements: “(1) I think I can be easily infected by HPV” (Mean score: 2.24; SD: 0.96); “(4) People with only one sexual partner have a low risk of getting infected with HPV” (Mean score: 2.64; SD: 1.01); “(11) Women are embarrassed to get a Pap test” (Mean score: 2.91; SD: 1.00); and “(20) I need extra information about the efficacy of the vaccine” (Mean score: 2.14; SD: 0.80) (Table 4).

Statistically significant gender differences were observed for four statements, but the differences of the scores were smaller than 0.5, which cannot be considered clinically significant. The statements were: “(8) Cervical cancer is not a big problem for women” (*p* < 0.05); “(11) Women are embarrassed to get a Pap test” (*p* < 0.05); “(12) HPV vaccination is not necessary because a Pap test can be done to rule out cervical cancer” (*p* < 0.01); and “(13) It is better to be vaccinated before becoming sexually active” (*p* < 0.05) (Table 4).

### 3.6. Knowledge of HPV Vaccination

Regarding knowledge of HPV vaccination, it was found that a high proportion of questions were answered incorrectly. In particular, there were five questions that received a correct response from less than one-third of the respondents. The questions were: “(1) More than 50% of sexually active women in Hong Kong have been infected with HPV once in their life” (14% correct); “(2) More than 50% of sexually active men in Hong Kong have been infected with HPV once in their life” (8.9% correct); “(7) Having a single sexual partner can prevent HPV infection” (24.9% correct); “(12) HPV that causes cervical cancer and genital warts is the same type” (30% correct); and “(15) Women over the age of 50 should start to get a Pap test” (26.5%) (Table 5).

Significant gender differences were seen in the responses to the following four questions: “(18) Women who were vaccinated against HPV do not need to get a Pap test” (males 60.1% *vs.* females 70.1%, *p* < 0.05); “(20) It is good to get vaccinated against HPV after becoming sexually active” (males 47.8% *vs.* females 61.5%, *p* < 0.01); “(21) The HPV vaccine is available in Hong Kong now” (males 80.3% *vs.* females 88.0%, *p* < 0.05); and “(22) The HPV vaccine can provide 100% protection against HPV-related diseases” (males 75.4% *vs.* females 83.8%, *p* < 0.05) (Table 5).

### 3.7. Predictors of the Respondents’ Attitude towards HPV Vaccination

After the univariate analysis, a multivariate linear regression analysis was performed on those variables that correlated with the respondents’ total score for attitude in the bivariate analysis with a *p*-value of smaller than 0.1. The final regression model included 10 predictors. Three of them were on gender, the acceptable maximum price of the vaccine when the efficacy is 100% for cervical cancer and genital warts, and the willingness to receive the HPV vaccine if it can protect against cervical/anal cancer and genital warts. The regression coefficients showed that female students, students willing to receive the HPV vaccine, and students who would pay a higher acceptable maximum price were likely to have a better attitude towards HPV vaccination. The remaining seven predictors were about the correctness of answering questions related to the HPV vaccine. All seven predictors indicated that students who correctly answered the questions had a better attitude towards HPV vaccination. The overall adjusted R^2^ of the regression model was 0.33, which was considered large (Table 6) [35]. The variance inflation factor (VIF) of all of the variables was less than 10, which suggested that there was no co-linearity problem [36].

## 4. Discussion

### 4.1. Low HPV Vaccination Uptake Compared to Similar Studies

In this study, the HPV vaccination rate of the Hong Kong undergraduates was estimated to be 13.3%. This is significantly lower that found than in some developed countries like Portugal and the United Kingdom, at about 80% and 81%, respectively [37]. This phenomenon could be related to vaccination subsidies, as countries that have set up an HPV vaccination program offering the vaccine at a low cost would have a high vaccination rate. This is also consistent with the result of this study, which found that most people would prefer to pay less than HK$500 to be vaccinated, regardless of the efficacy of the vaccine. A lower price might increase the intention to get vaccinated. Furthermore, the vaccination rate in this study is consistent with that found in studies conducted in countries that do not have a vaccination subsidy program. For example, Bang *et al.* found that 12% of university students in Seoul had been vaccinated against HPV [38]. To raise the HPV vaccination rate, the government may need to consider subsidizing the vaccination.

### 4.2. Intention of Vaccination

In this study, the figure for students who reported that they intended to get vaccinated against HPV was 69.6%, which is considered modest. A similar study conducted in Korea reported a similar HPV vaccination intention of 62.8% [28]. This modest intention was probably related to a relative knowledge deficit, as more knowledge increases the intention to be vaccinated. Another reason why young adults might be reluctant to receive the HPV vaccine to prevent sexually transmitted diseases is that young adults may be afraid of being labeled sexually active. A previous Asian study reported that young women were worried that getting vaccinated against HPV would give others the impression that they were sexually active [39]. On the other hand, Giuseppe *et al.* in Italy reported that a higher intention to receive HPV vaccination was associated with more openness to sex-related topics [40].

### 4.3. Knowledge of HPV and HPV Vaccination

In general, the knowledge of undergraduate students regarding HPV and HPV vaccination was poor. The mean percentage of correct answers to questions about HPV was only 52.7% in this study, which was lower than a previous study conducted in the U.S. [41]. Dillard and Spear interviewed 396 undergraduate female students aged 18–24 and reported a mean correct knowledge score of 65%. The relatively low level of knowledge is probably related to the little coverage that is given to HPV in sex education programs in Hong Kong. This study also showed that students studying in programs unrelated to healthcare were able to answer fewer questions correctly compared to those in healthcare-related disciplines. Students studying subjects related to healthcare have learned about cervical cancer and would have acquired more knowledge about the causes of such diseases as cancer. The significantly higher scores of students studying healthcare-related disciplines might imply that better education is effective at improving the knowledge and attitudes of students about the uptake of HPV vaccination.

### 4.4. Predictors of Students’ Attitude towards HPV Vaccination

In this study, the most significant positive predictors of the students’ attitude towards HPV vaccination were those related to their knowledge of HPV and HPV vaccination. Seven out of ten significant predictors identified from the regression analysis were related to the knowledge test (Table 6). Janz and Backer noted that high perceived susceptibility, perceived severity, and perceived benefits may trigger health-promoting behaviors and change people’s attitude towards taking health-related actions. Students with poorer knowledge about HPV infection and vaccination are believed to have a lower perception of the risk and severity of HPV infection, and a lower perception of the benefits of HPV vaccination [42]. According to the Health Belief Model [43], a poorer level of knowledge among the respondents may indicate a lower intention of getting vaccinated against HPV. This finding explained the low vaccination rate of the respondents in the study. In contrast, a study showed that the intention to get vaccinated increased from 35% to 69% after the participants read an information pamphlet on the subject [44]. Knowledge is an important predicator of vaccination rates. On the other hand, in the final regression model there was a significant predictor related to acceptability, which was the maximum acceptable price of the HPV vaccine. In addition to the level of knowledge about the vaccine, it is also important to keep the price of the vaccine at an affordable level in relation to its efficacy.

Among the male students, the injection rate of the HPV vaccine was only 0.5%. The results of the regression analysis also indicate that gender is a significant factor affecting attitudes toward HPV vaccination. The health promotion efforts on preventing HPV infection have mainly focused on cervical cancer, which is not related to the health of males. Male students may not know about the connection between HPV infection and genital warts. With a lower level of knowledge, they may be less aware of the full potential impact of HPV infection to themselves as males, and the benefits of vaccination.

### 4.5. Limitations

First, as convenience sampling was used and all of the respondents were from the same university, the results may not be representative enough for the findings to be generalizable to the entire population [45]. Students with family history of cervical cancer or other HPV-related health issues were not excluded from the study, which may contaminate the results because they are expected to have a different belief and behavioral pattern on HPV vaccination. Another limitation of this study was that only the English version of the questionnaires was provided to the participants. There were some medical terms such as the Pap test, sexology, and genital warts, which may have been difficult for students in disciplines unrelated to healthcare to understand, and this may have led to some misunderstanding of the questions and affected results of the study. Furthermore, the questionnaires did not include questions related to homosexual relationships. A study by Gutierrez *et al.* found that men who have sex with men (MSM) are at a higher risk of contracting HPV than heterosexual men, and a higher perceived susceptibility among this group should be anticipated. The study also found that MSM were more capable of getting the HPV vaccine than heterosexual men [46]. In addition, the question on relationship status could be adjusted by including more levels such as married and divorced. Questions such as that on the number of sexual partners could also be included to obtain a better understanding of the information relating to the sexual experience of the participants. Moreover, questions relating to the three-dose requirement for HPV and whether this is a barrier should also be included. Finally, this study only examined the attitudes of the participants toward HPV vaccination, but their intention may not reflect their actual practice or behaviors.

### 4.6. Recommendations

This study found that male undergraduate students have a lower perception of the risks of HPV infection, which may be due to a knowledge deficit. Public health professionals and policy makers should conduct better health promotion efforts in universities based on the factors and knowledge deficit that have been identified, in order to increase HPV vaccination among undergraduate students. Promotion of HPV vaccination may be delivered through an online platform or on television, which, according to the results of this study, are the two most common channels through which information about HPV is acquired. The health promotion activities should also focus more on male vaccination, due to the very low male vaccination rate. Emphasizing the link between HPV infections and genital warts may draw more attention from males than emphasizing cervical cancer only. The government might also provide some financial subsidies, which could increase the willingness of students to get vaccinated. A vaccination promotion program and registry should be established, thus providing a more reliable source of HPV vaccination rate and statistics. Further studies could be carried out to investigate factors affecting the attitudes towards vaccination of people in homosexual relationships, e.g., MSM.

## 5. Conclusions

The knowledge and awareness of local undergraduate students in Hong Kong concerning HPV infection is not high and their HPV vaccination rate is relatively low. More aggressive education and promotion activities on HPV and HPV vaccination should be organized for undergraduates, especially for male students, as their knowledge and attitude towards the subject is poorer than that of female students. Awareness of HPV infection should be promoted among male students.

## Figures and Tables

**Table 1:** Socio-demographic characteristics.

Variable		*n* (%)
Gender	Male	203 (46.5)
	Female	234 (53.5)
Program	Related to healthcare	232 (53.1)
	Not related to healthcare	205 (46.9)
Age	18–21	291 (66.6)
	22–25	146 (33.4)
Monthly Income	<HK$3000	294 (67.3)
	HK$3001–$6000	107 (24.5)
	HK$6001–$9000	16 (3.7)
	>HK$9000	20 (4.6)
Residence Status	Live with family	395 (90.4)
	Shared Dormitory	31 (7.1)
	Living Alone	7 (1.6)
	Others	4 (0.9)
Religion	Buddhism	18 (4.1)
	Catholicism	9 (2.1)
	Christianity	93 (21.4)
	Others	1 (0.2)
	None	314 (72.2)
Relationship Status	Single	276 (63.2)
	In a relationship with 1 person	159 (36.4)
	In a relationship with >1 person	2 (0.5)
Had Sexual Experience		73 (17.0)

**Table 2:** Medical background.

Variable		*n* (%)
History of genital warts		2 (0.5)
History of anal cancer		1 (0.2)
History of cervical cancer		0 (0)
Heard about cervical cancer		384 (87.9)
Source of information about cervical cancer	Television	322 (73.7)
	Newspapers/Magazines	187 (42.8)
	Internet	184 (42.1)
	Healthcare Provider	176 (40.3)
	Friend/Relative	110 (25.2)
	Radio	110 (25.2)
	Others	45 (10.3)
Heard about the HPV vaccine		403 (92.2)
Source of information about the HPV vaccine	Television	330 (75.5)
	Internet	191 (43.7)
	Newspapers/Magazines	177 (40.5)
	Healthcare Provider	176 (40.3)
	Friend/Relative	133 (30.4)
	Radio	111 (25.4)
	Others	52 (11.9)
History of vaccination	Female	57 (24.4)
	Male	1 (0.5)
	Total	58 (13.3)
Willingness to receive the HPV vaccine if it can protect against cervical/anal cancer and genital warts	Would like to be vaccinated	300 (69.6)
	Would not like to be vaccinated	84 (19.5)
	Cannot make a decision	47 (10.9)

**Table 3:** Students’ acceptance of HPV vaccination.

Variables		*n* (%)
If the HPV vaccine is available and deliverable in the places listed below, which option would make it more likely that you would go to get vaccinated against HPV?	Health clinics in universities	189 (43.2)
	Public hospitals	149 (34.1)
	General practitioners	97 (22.2)
	Private hospitals	63 (14.4)
If the HPV vaccine can protect against both cervical cancer and genital warts, who should get the vaccination?	Both males and females should get vaccinated equally	339 (77.6)
	Females should get it rather than males ^+^	68 (15.6)
	Females only	28 (6.4)
	Males only	2 (0.5)
	Males should get it rather than females ^++^	0 (0)
If the HPV vaccine is beneficial for adolescents who are not sexually active, which of the following would be the ideal age for vaccination?	15–16 years old	117 (26.8)
	17–18 years old	97 (22.2)
	13–14 years old	95 (21.7)
	11–12 years old	79 (18.1)
	≤10 years old	45 (10.3)
	>18 years old	4 (0.9)

^+^ This option means that getting vaccinated is much more important for females than males. ^++^ This option means that getting vaccinated is much more important for males than females.

**Table 4:** Gender differences in attitude towards HPV vaccination.

Questions on Attitude towards HPV Vaccination	Males	Females	Total	*t*-Statistics
	Mean (SD)	Mean (SD)	Mean (SD)	
(1) I think I can be easily infected by HPV	2.15 (1.02)	2.32 (0.89)	2.24 (0.96)	−1.84
(2) I think HPV infection is a serious disease	4.03 (0.87)	4.04 (0.73)	4.04 (0.80)	−0.05
(3) Preventing HPV infection is important for women	4.25 (0.78)	4.36 (0.71)	4.31 (0.74)	−1.64
(4) People who have only one sexual partner have a low risk of becoming infected with HPV	2.55 (1.02)	2.71 (0.99)	2.64 (1.01)	−1.65
(5) Using a condom can provide 100% protection against HPV infection	3.93 (0.96)	4.06 (0.92)	4.00 (0.94)	−1.44
(6) Preventing HPV infection is important for men	3.72 (0.85)	3.72 (0.86)	3.72 (0.85)	−0.04
(7) Education on HPV should be implemented at school	4.12 (0.81)	4.26 (0.80)	4.19 (0.80)	−1.86
(8) Cervical cancer is not a big problem for women	4.11 (0.99)	4.34 (0.88)	4.23 (0.93)	−2.52 *
(9) Cervical cancer may cause death for women	4.03 (0.86)	4.18 (0.81)	4.11 (0.84)	−1.87
(10) Men can get involved to prevent their partner from getting cervical cancer	3.88 (0.83)	3.91 (0.83)	3.89 (0.83)	−0.31
(11) Women are embarrassed to get a Pap test	2.79 (0.96)	3.02 (1.03)	2.91 (1.00)	−2.34 *
(12) HPV vaccination is not necessary because a Pap test can be done to rule out cervical cancer	3.56 (0.95)	3.81 (0.91)	3.70 (0.93)	−2.76 **
(13) It is better to be vaccinated before becoming sexually active	3.85 (0.87)	4.04 (0.85)	3.95 (0.86)	−2.31 *
(14) It is preferable to vaccinate both men and women	3.96 (0.82)	4.04 (0.77)	4.00 (0.79)	−1.03
(15) I am well enough informed to request vaccination without parental consent	3.33 (0.95)	3.43 (0.90)	3.38 (0.92)	−1.08
(16) I have sufficient information about HPV and its vaccine to decide whether to receive the vaccine	3.08 (0.98)	3.18 (0.86)	3.13 (0.92)	−1.15
(17) Hong Kong adolescents need sex education	3.80 (0.82)	3.84 (0.84)	3.82 (0.83)	−0.44
(18) I am sure that the HPV vaccine is highly effective	3.40 (0.77)	3.43 (0.75)	3.42 (0.76)	−0.32
(19) I am sure that the HPV vaccine is safe	3.40 (0.82)	3.40 (0.80)	3.40 (0.81)	0.08
(20) I need extra information about the efficacy of the HPV vaccine	2.15 (0.88)	2.13 (0.73)	2.14 (0.80)	0.31
(21) HPV vaccination can lead to an increase in risky sexual behavior	3.24 (0.89)	3.38 (0.87)	3.32 (0.88)	−1.76

* *p* < 0.05; ** *p* < 0.01.

**Table 5:** Gender differences in knowledge of HPV vaccination.

Question Type	Questions on HPV and HPV Vaccination	Correctly Answered			χ^2^
		Males	Females	Total	
		*n* (%)	*n* (%)	*n* (%)	
Questions on HPV	(1) More than 50% of sexually active women in Hong Kong have been infected with HPV once in their life	34 (16.7)	27 (11.5)	61 (14.0)	2.46
	(2) More than 50% of sexually active men in Hong Kong have been infected with HPV once in their life	21 (10.3)	18 (7.7)	39 (8.9)	0.94
	(3) You get HPV infection only through having sex.	140 (69.0)	166 (70.9)	306 (70.0)	0.20
	(4) Using condoms can completely prevent HPV infections	151 (74.4)	183 (78.2)	334 (76.4)	0.88
	(5) HPV can cause cervical cancer	153 (75.4)	175 (74.8)	328 (75.1)	0.02
	(6) Normally, you do not have any signs and symptoms when you get an HPV infection	80 (39.4)	95 (40.6)	175 (40.0)	0.06
	(7) Having a single sexual partner can prevent HPV infection	51 (25.1)	58 (24.8)	109 (24.9)	0.01
	(8) HPV can cause genital warts	117 (57.6)	119 (50.9)	236 (54.0)	2.01
	(9) HPV infection occurs only in women	149 (73.4)	181 (77.4)	330 (75.5)	0.92
	(10) Early sexual activity is a risk factor for HPV infection	101 (49.8)	123 (52.6)	224 (51.3)	0.34
	(11) A Pap test is a kind of cervical cancer treatment	96 (47.3)	125 (53.4)	221 (50.6)	1.63
	(12) The HPV that causes cervical cancer and genital warts is of the same type	64 (31.5)	67 (28.6)	131 (30.0)	0.43
	(13) Cervical cancer can be detected in the early stage	142 (70.0)	153 (65.4)	295 (67.5)	1.03
	(14) Persistent infections of HPV can cause cervical cancer	113 (55.7)	126 (53.8)	239 (54.7)	0.15
	(15) Women over the age of 50 should start to get a Pap test	49 (24.1)	67 (28.6)	116 (26.5)	1.13
Questions on HPV vaccination	(16) There is a vaccine to protect against cervical cancer and genital warts	117 (57.6)	141 (60.3)	258 (59.0)	0.31
	(17) The HPV vaccine is currently recommended for women only	124 (61.1)	142 (60.7)	266 (60.9)	0.01
	(18) Women who were vaccinated against HPV do not need to get a Pap test	122 (60.1)	164 (70.1)	286 (65.4)	4.79 *
	(19) The HPV vaccine is recommended for males and females of every age group	71 (35.0)	89 (38.0)	160 (36.6)	0.44
	(20) It is good to get vaccinated against HPV after becoming sexually active	97 (47.8)	144 (61.5)	241 (55.1)	8.32 **
	(21) The HPV vaccine is available in Hong Kong now	163 (80.3)	206 (88.0)	369 (84.4)	4.95 *
	(22) The HPV vaccine can provide 100% protection against HPV-related diseases	153 (75.4)	196 (83.8)	349 (79.9)	4.76 *

* *p* < 0.05; ** *p* < 0.01.

**Table 6:** Predictors of the respondents’ attitude towards receiving the HPV vaccine.

Predictors		B	95% CI	*t*-Value	VIF	adj-R^2^
(1) Gender (0: female; 1: male)		−1.40	(−2.48, −0.33)	−2.57 *	1.05	0.33
(2) Acceptable maximum price of the vaccine when the efficacy is 100% for cervical cancer and genital warts (1: <$500; 7: >$1500)		0.35	(0.04, 0.66)	2.22 *	1.07	
(3) Willingness to receive the HPV vaccine if it can protect against cervical/anal cancer and genital warts (1: I would like to be vaccinated; 2: I cannot make a decision; 3: I would not like to be vaccinated)		−1.90	(−2.57, −1.24)	−5.60 ***	1.04	
Knowledge of vaccination	(4) HPV infection occurs only in women (0: incorrect or don’t know; 1: correct)	2.18	(0.78, 3.58)	3.06 **	1.31	
	(5) Persistent HPV infections can cause cervical cancer (0: incorrect or don’t know; 1: correct)	1.90	(0.74, 3.07)	3.22 **	1.23	
	(6) The use of condoms can completely prevent HPV infection (0: incorrect or don’t know; 1: correct)	2.30	(1.01, 3.60)	3.49 **	1.09	
	(7) The HPV vaccine is currently recommended only for women (0: incorrect or don’t know; 1: correct)	1.94	(0.72, 3.17)	3.12 **	1.31	
	(8) It is good to get vaccinated against HPV after becoming sexually active (0: incorrect or don’t know; 1: correct)	1.76	(0.65, 2.87)	3.12 **	1.12	
	(9) More than 50% of sexually active women in Hong Kong have been infected with HPV once in their life (0: incorrect or don’t know; 1: correct)	2.09	(0.56, 3.61)	2.69 **	1.05	
	(10) There is a vaccine to protect against cervical cancer and genital warts (0: incorrect or don’t know; 1: correct)	1.36	(0.19, 2.53)	2.29 *	1.20	

* *p* < 0.05; ** *p* < 0.01; *** *p* < 0.001.

## Abbreviations

The following abbreviations are used in this manuscript:

HPV	Human Papillomavirus
MSM	Men who have sex with other men
